# Establishment of *Agrobacterium*-Mediated Transient Transformation System in Sunflower

**DOI:** 10.3390/plants14152412

**Published:** 2025-08-04

**Authors:** Fangyuan Chen, Lai Wang, Qixiu Huang, Run Jiang, Wenhui Li, Xianfei Hou, Zihan Tan, Zhonghua Lei, Qiang Li, Youling Zeng

**Affiliations:** 1 Xinjiang Key Laboratory of Biological Resources and Genetic Engineering, College of Life Science and Technology, Xinjiang University, Urumqi 830017, China; chenfangyuan@stu.xju.edu.cn (F.C.); 13579285361@163.com (L.W.); runjiang@stu.xju.edu.cn (R.J.); liwenhuixjust@163.com (W.L.); tanzy1028@163.com (Z.T.); 2 Crop Research Institute, Xinjiang Uygur Autonomous Region Academy of Agricultural Sciences, Urumqi 830091, China; huangqx@xaas.ac.cn (Q.H.); houxianfei@xaas.ac.cn (X.H.); leizh@xaas.ac.cn (Z.L.); 3 Key Laboratory of Microbial Resources Protection, Development and Utilization, College of Biological Sciences and Technology, Yili Normal University, Yining 835000, China; 4 College of Medicine, Xinjiang College of Science & Technology, Korla 841000, China

**Keywords:** sunflower, transient transformation, *Agrobacterium*-mediated, infiltration, injection, ultrasonic-vacuum, establishment and optimization

## Abstract

Sunflower (*Helianthus annuus* L.) is an important oilseed crop in Northwest China, exhibiting resistance to salt and drought. Mining its excellent tolerance genes can be used for breeding. However, the current platforms for identifying gene function in sunflower is inadequate. The transient transformation system, which can rapidly validate gene function, shows promising prospects in research. In this study, we established an efficient transient expression transformation system for sunflower using three methods: *Agrobacterium*-mediated infiltration, injection, and ultrasonic-vacuum. The detailed procedures were as follows: *Agrobacterium* GV3101 carrying a *GUS* reporter gene on the pBI121 vector with an OD_600_ of 0.8 as the bacterial suspension and 0.02% Silwet L-77 as the surfactant were utilized in all three approaches. For the infiltration method, seedlings grown hydroponically for 3 days were immersed in a bacterial suspension containing 0.02% Silwet L-77 for 2 h; for the injection method, the same solution was injected into the cotyledons of seedlings grown in soil for 4 to 6 days. Subsequently, the seedlings were cultured in the dark at room temperature for three days; for the ultrasonic-vacuum method, seedlings cultured in Petri dishes for 3 days were first subjected to ultrasonication at 40 kHz for 1 min, followed by vacuum infiltration at 0.05 kPa for 5–10 min. *Agrobacterium*-mediated transient transformation efficiency achieved by the three methods exceeded 90%, with gene expression being sustained for at least 6 days. Next, we employed the infiltration-based sunflower transient transformation technology with the *Arabidopsis* stable transformation platform to confirm salt and drought stress tolerance of candidate gene *HaNAC76* from sunflower responding to various abiotic stresses. Altogether, this study successfully established an *Agrobacterium*-mediated transient transformation system for sunflower using these three methods, which can rapidly identify gene function and explore the molecular mechanisms underlying sunflower’s resistance traits.

## 1. Introduction

The sunflower (*Helianthus annuus* L.) is an important oil crop, contributing 9.0% to global edible oil production [1]. It also has high salt tolerance and is known as “a pioneer crop for saline soil” [2]. Mining of sunflower resistance genes are crucial for breeding varieties with superior traits. In fact, the sequencing of the sunflower genome was completed in 2017 [3], but the work of relying on stable genetic transformation technology to identify gene functions has advanced slowly. This is mainly because the application of this technology in most plants is limited due to genotype dependence, regeneration difficulties, and low transformation efficiency [4], and sunflower suffers from these problems as well [5]. Only one previous study reported that merely 20 heritable-positive plants in T_4_ generation were obtained from a total of 24,328 explants through stable transformation using the sunflower restorer line (RHA 6D-1) genotype [6]. However, in the current context, relying solely on genetic transformation in model plants such as *Arabidopsis thaliana*, tobacco, or other species for gene function validation is no longer sufficient [7,8]. Instead, in situ confirmation has become increasingly essential. Therefore, there is an urgent necessity to establish a homologous expression system in sunflower to comprehensively analyze gene functions and the biological process involved.

For species that have not yet established a stable transformation system, transient transformation technology can serve as an effective tool to rapidly identify gene functions with short time and low cost [9,10,11]. With the development of this technology, currently reported methods include *Agrobacterium*-mediated gene transformation, particle bombardment, electroporation, and polyethylene glycol-mediated transformation [12,13,14,15,16,17]. Among them, *Agrobacterium*-mediated gene transformation is widely preferred for its high transfer efficiency and simplicity [18]. The principle involves first transforming the vector containing the target gene into *Agrobacterium*. Subsequently, the gene within the bacterial infection buffer is introduced into plant cells via various methods to achieve transient expression. Actually, this entire process requires only a few days [19]. This method can be applied to a wide range of tissues, including leaves, petals, petioles, hypocotyls, callus, stem segments, and fruit pulp [20,21,22,23,24,25]. Additionally, it is feasible to immerse entire plants using this method [26,27,28]. Examples of gene function validation using transient systems have been reported: transient overexpression of chalcone synthase gene *CtCHS1* in safflower (*Carthamus tinctorius*) callus significantly induced an increase in the content of naringenin and genistein in the flavonoid biosynthetic pathway [29]; similarly, transient overexpression of dehydration-responsive element binding protein 1C (*CiDREB1C*) in the leaves of *Caragana intermedia* enhanced the plant tolerance to salt and drought stresses, while simultaneously reducing its sensitivity to abscisic acid [30]; and transient overexpression of the *SgCPR1/2* gene involved in the mogroside biosynthesis pathway in the fruit of monk fruit (*Siraitia grosvenorii*) enhanced the production of the key secondary metabolites [31]. Therefore, the establishment and application of a transient transformation system in sunflower are both feasible and necessary.

Expression of stress responsive genes is induced when plants are exposed to abiotic stresses. The NAC (NAM/ATAF/CUC) family is one of the largest groups of plant-specific transcription factors, with many members playing a role in regulating abiotic stress tolerance in plants. *MdNAC29* reduces drought tolerance in apple by regulating the expression of genes related to photosynthesis and leaf senescence [32]; *GmNAC12* positively regulates drought stress tolerance in soybeans by improving the antioxidant activities [33]. In our laboratory, sunflower *NAC76* (*HaNAC76*) has been demonstrated to respond to a variety of abiotic stress. However, its functional roles and the underlying stress tolerance mechanisms remain to be elucidated.

To establish and optimize a transient transformation system for sunflower, we used β-glucuronidase (GUS) as a reporter gene to compare and optimize three *Agrobacterium*-mediated methods: infiltration, injection, and ultrasonic-vacuum. Our results demonstrate that all three methods are suitable for transient transformation in sunflower. The salt and drought tolerance of *HaNAC76* was fully investigated through infiltration-based transient transformation in sunflower and stable transformation in *Arabidopsis*. This transient transformation platform enables the rapid and effective identification of gene functions and facilitates a broad range of molecular biology experiments in sunflower. The application of this system holds great potential for advancing our understanding of the mechanisms underlying sunflower stress resistance.

## 2. Results

### 2.1. Optimization for Transient Transformation with the Infiltration Method in Sunflower

**Figure 1:**
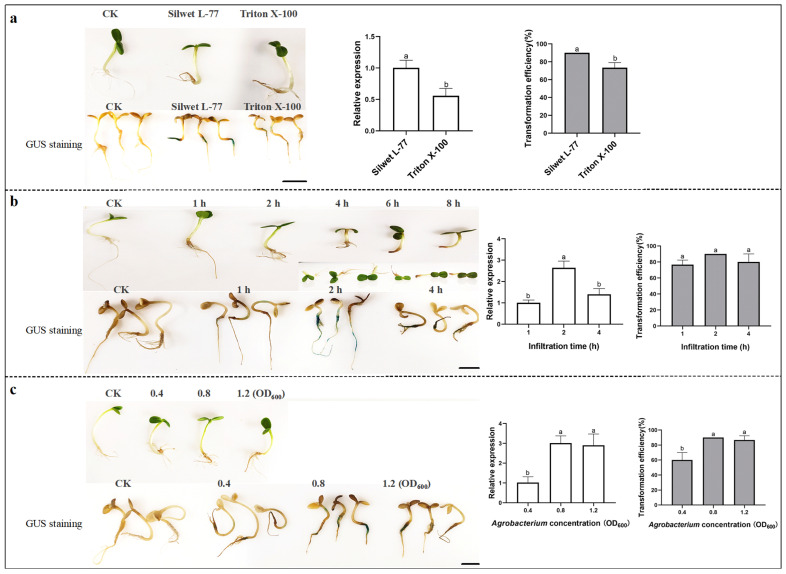
Optimization of transient transformation in sunflower using the infiltration method, evaluated through three aspects: GUS staining of seedlings, relative *GUS* expression levels, and transformation efficiency. Three factors were investigated for their effects on transformation efficiency: (**a**) surfactants (Silwet L-77 and Triton X-100); (**b**) infiltration times (1 h, 2 h, 4 h, 6 h, and 8 h); and (**c**) *Agrobacterium* concentration (OD_600_: 0.4, 0.8, and 1.2). The data were analyzed using one-way analysis of variance (ANOVA), with treatment means presented as mean ± standard deviation (SD). Different lowercase letters indicated significant differences in the detected values of various types of treatments (Tukey’s least significant difference (LSD) test was used for comparison at *p* < 0.05). Scale bars: 1.0 cm.

To identify the most effective transformation parameters with the infiltration method in sunflower, three key factors were evaluated: surfactant type, infiltration time, and *Agrobacterium* concentration. Surfactants play an important role in promoting bacterial invasion of plant tissues, and their effects can vary significantly across different plant species [34]. In this study, we compared two surfactants: Silwet L-77 and Triton X-100. Seedlings soaked in an infiltration solution containing Silwet L-77 exhibited more pronounced GUS staining and a significantly higher level of *GUS* gene expression, with a 44.4% increase compared to those treated with Triton X-100. The positive seedling transformation rate reached 90% when Silwet L-77 was used (Figure 1a). The infiltration time was another critical factor. Prolonged immersion of seedlings in the infiltration solution, which primarily consisted of sucrose, caused tissue and organ damage, particularly to the root system. After 4 h of immersion, partial root necrosis was observed, and complete root necrosis occurred after 6–8 h. Seedlings immersed for 1 h showed light GUS staining and the lowest relative gene expression. Based on these observations, the optimal infiltration time for sunflower was determined to be 2 h (Figure 1b). The *Agrobacterium* concentration also significantly affected the transformation efficiency. At an OD_600_ of 0.4, GUS staining was light, the relative expression of the *GUS* gene was low, and the transformation efficiency was only 60%. In contrast, at an OD_600_ of 0.8, the infiltration effect was optimal, with both *GUS* gene expression and transformation efficiency reaching 90% (Figure 1c). Totally, the optimal parameters for transient transformation of sunflower using the infiltration method were as follows: an *Agrobacterium* concentration with an OD_600_ of 0.8, the addition of Silwet L-77 as a surfactant, and an infiltration time of 2 h.

### 2.2. Optimization for Transient Transformation with the Injection in Sunflower

**Figure 2:**
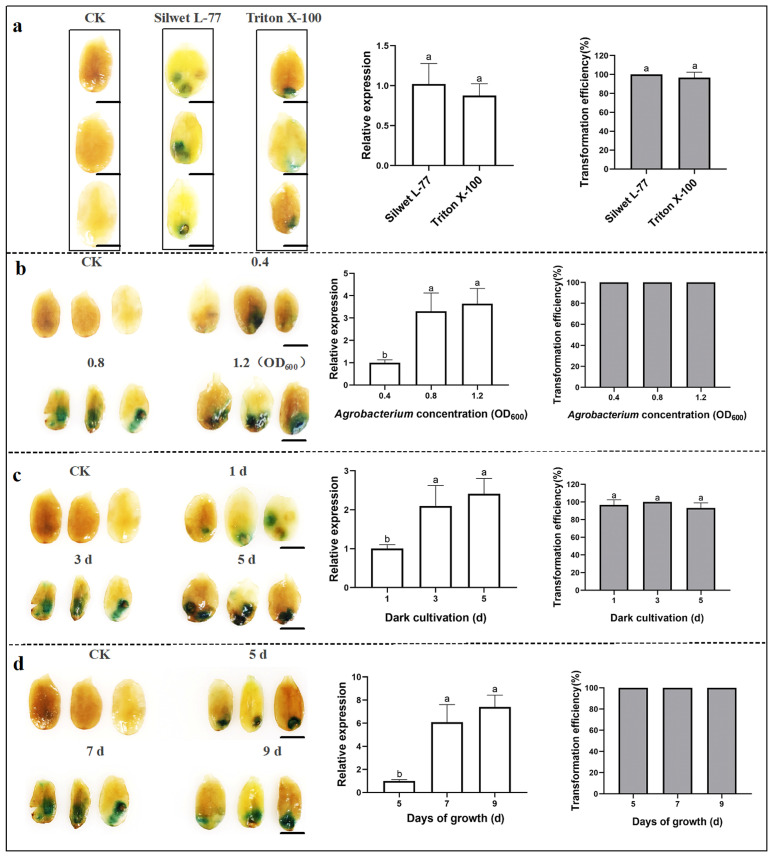
Optimization of transient transformation in sunflower using the injection method, evaluated through GUS staining of seedlings, relative *GUS* expression levels, and transformation efficiency. Four factors were tested for their impact on transformation efficiency: (**a**) surfactants (Silwet L-77 and Triton X-100); (**b**) *Agrobacterium* concentration (OD_600_: 0.4, 0.8, and 1.2); (**c**) dark cultivation durations (1 d, 3 d, 5 d); (**d**) cotyledon developmental stages (5 d, 7 d, and 9 d). The experimental data were analyzed by ANOVA. Differences in treatment means were presented as mean ± SD. Different lowercase letters indicated significant differences in the detected values of various types of treatments (Tukey’s least significant difference (LSD) test was used for comparison at *p* < 0.05). Scale bars: 0.5 cm.

The injection method involved direct delivery of an *Agrobacterium* suspension into plants using a syringe. To establish the optimal transformation conditions for this technique, four parameters were evaluated: surfactant type, *Agrobacterium* concentration, dark cultivation time, and seedling growth stage. The optimal transformation efficiency was achieved using Silwet L-77 as the surfactant and an *Agrobacterium* concentration with an OD_600_ of 0.8, which were aligned with the optimal results using the infiltration method (Figure 2a). Among these conditions, the most intense GUS staining was observed at an *Agrobacterium* concentration with an OD_600_ of 1.2, with a 72.92% increase in relative *GUS* expression compared to that at an OD_600_ of 0.4. However, the cotyledon in the injection area was almost necrotic under this condition. In contrast, at an *Agrobacterium* concentration with an OD_600_ of 0.8, the relative *GUS* expression increased by 69.52% compared to that at an OD_600_ of 0.4, with smaller damage in cotyledon. Therefore, an *Agrobacterium* concentration with an OD_600_ of 0.8 was determined to be the optimal (Figure 2b). Dark cultivation promoted expression of exogenous genes introduced by *Agrobacterium* in sunflower seedlings. Lighter GUS staining and lower relative gene expression were observed after 1 day of dark cultivation. However, after 5 days of dark cultivation, cotyledons became necrotic due to prolonged “starvation” and excessive *Agrobacterium* infection. Thus, 3 days of dark cultivation was determined to be optimal with a 56.5% increase in relative *GUS* gene expression compared to that after 1 day of dark culture (Figure 2c). Seedlings at different stages can also affect the efficiency of infection. Newly unfolded cotyledons on day 5 were the least suitable for injection due to tight adherence between the cotyledon epidermis and the underlying mesophyll tissue. In contrast, seedlings grown for 7–9 days exhibited improved infection, deeper GUS staining, and an average increase of 88.0% in relative *GUS* gene expression in both groups (Figure 2d). Collectively, the optimal transient transformation conditions using the injection method were as follows: Silwet L-77 as the surfactant, an *Agrobacterium* concentration with an OD_600_ of 0.8, 3 days of dark cultivation, and a seedling growth stage of 7 to 9 days.

### 2.3. Optimization for Transient Transformation Using Ultrasonic-Vacuum in Sunflower

**Figure 3:**
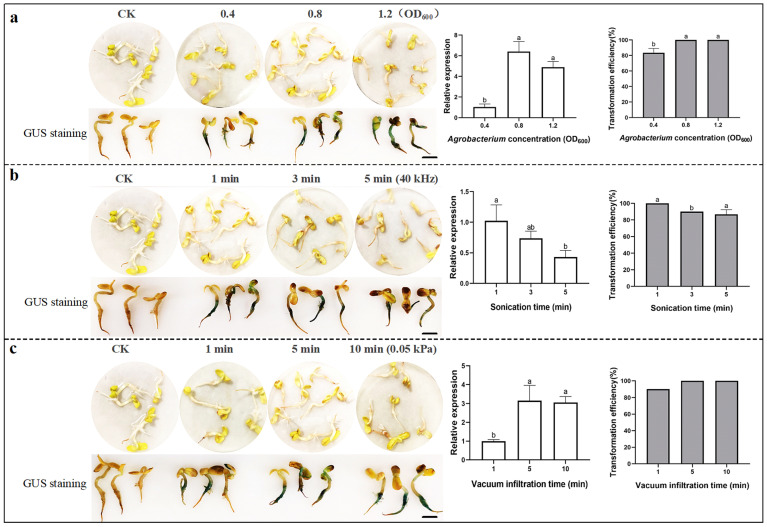
Optimization of transient transformation in sunflower using the ultrasonic-vacuum method, evaluated through GUS staining of seedlings, relative *GUS* expression level and transformation efficiency. Three factors were tested for their impact on transformation efficiency: (**a**) *Agrobacterium* concentration (OD_600_: 0.4, 0.8, and 1.2); (**b**) ultrasonic time (1 min, 3 min, and 5 min); (**c**) vacuum filtration time (1 min, 5 min, and 10 min). The experimental data were analyzed by ANOVA. Differences in treatment means were expressed as mean ± SD. Different lowercase letters indicated significant differences in the detected values of various types of treatments (Tukey’s least significant difference (LSD) test was used for comparison at *p* < 0.05). Scale bars: 1.0 cm.

The ultrasonic-vacuum method employs an ultrasonic cleaner to create microscopic wounds and vacuum filtration to enhance *Agrobacterium* infection for sunflower seedling. The relative expression of *GUS* genes increased by 87.1% under an *Agrobacterium* concentration with an OD_600_ of 0.8 compared to under that with an OD_600_ of 0.4 (Figure 3a). However, cotyledon necrosis was observed when ultrasonication exceeded 3 min (Figure 3b). Vacuum infiltration promoted large quantities of the target gene into plant cells, thereby significantly improving transformation efficiency. Seedlings achieved 100% transformation efficiency and the most intense GUS staining after 5–10 min of vacuum filtration, with a 66.67% increase in relative gene expression compared to the 1 min treatment (Figure 3c). In a word, the optimal conditions for transient transformation of sunflower using the ultrasonic-vacuum method were as follows: an *Agrobacterium* suspension with an OD_600_ of 0.8, ultrasonication at 40 kHz for 1 min, and vacuum infiltration with 0.05 kPa for 5–10 min.

### 2.4. Duration of Gene Expression in the Sunflower Transient Transformation System Using Three Methods

**Figure 4:**
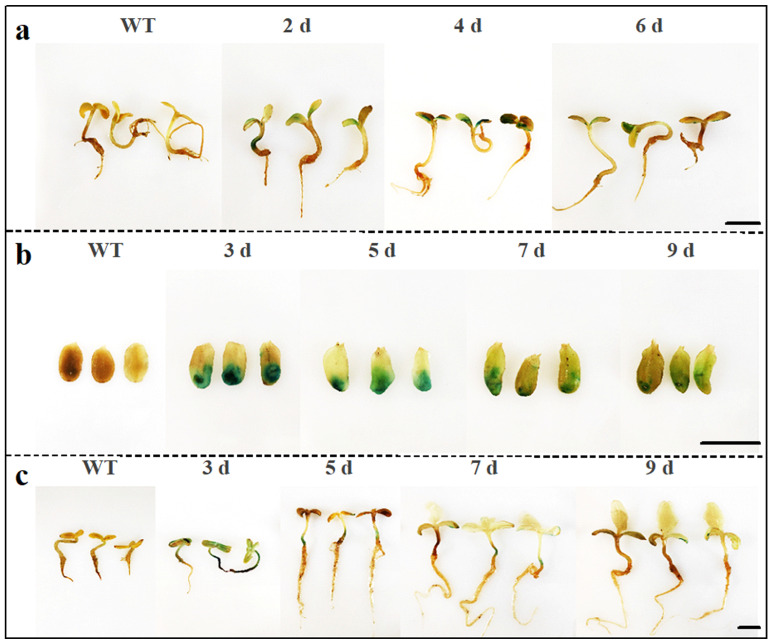
Duration of *GUS* gene expression in the sunflower transient transformation system. (**a**–**c**) GUS staining of sunflower seedlings transiently transformed was observed following treatments with infiltration and cultivation for 2–6 days, injection, and ultrasonic-vacuum combined with cultivation for 3–9 days. Scale bars: 1.0 cm.

To further evaluate gene expression using the transient expression transformation system of sunflower established using *Agrobacterium*-mediated infiltration, injection, and ultrasonic-vacuum techniques, GUS staining and analysis were performed. In the infiltration-based transient transformation method, seedlings were collected and analyzed after *Agrobacterium* infiltration and plant cultivation for 2, 4, and 6 days. *GUS* gene expression remained high from day 2 to day 4 and persisted until day 6 (Figure 4a). In the injection and ultrasonic-vacuum methods, cotyledons or seedlings were collected for GUS staining analysis at 3, 5, 7, and 9 days post-infiltration. GUS expression in both methods remained elevated from day 3 to day 5 and continued through day 9 (Figure 4b,c).

### 2.5. Functional Characterization of a Candidate Gene, HaNAC76 in Salt Tolerance and Drought Resistance by Agrobacterium-Mediated Transient Infiltration in Sunflower and Stable Genetic Transformation in Arabidopsis

**Figure 5:**
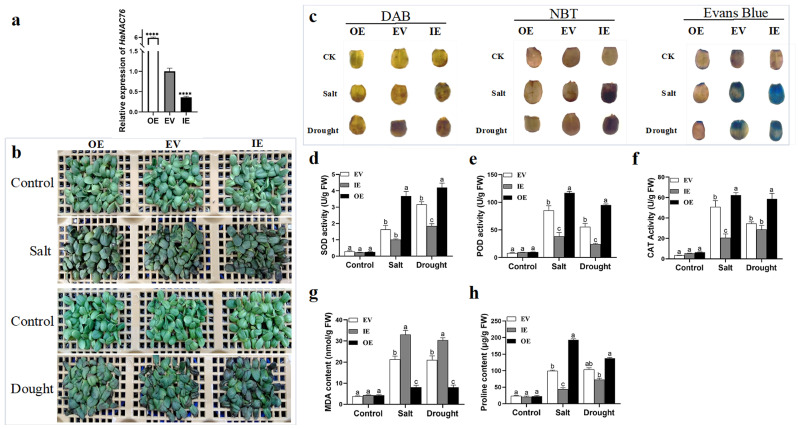
Characterization of the candidate gene *HaNAC76* for salt and drought tolerance in sunflower using *Agrobacterium*-mediated transient transformation by infiltration. (**a**) The growth phenotypes of different types of sunflower seedlings (OE, EV, and IE) under salt and drought stress. (**b**) The relative expression of *HaNAC76* detected by qRT-PCR in different types of sunflower seedlings before stress treatment. (**c**) DAB, NBT, and Evans Blue staining of the leaves of sunflower seedlings under stress conditions. (**d**–**h**) Determination of physiological indices in different types of sunflower seedlings, including activities of SOD, POD, and CAT, MDA content, and proline content under stress conditions. Statistical analyses were performed using Student’s *t*-test to compare differences in the relative expression of *HaNAC76* across different sunflower plants (**** *p* < 0.0001). Tukey’s test was employed to analyze between-group differences in physiological indices, with different lowercase letters indicating significant differences at the *p* < 0.05 level. Scale bars: 0.5 cm.

Three transient transformation methods in sunflower all showed good transformation results by comparing GUS staining of seedlings, relative *GUS* gene expression, and transformation efficiency. Here, a transient method by infiltration was employed to characterize the function of a candidate gene, *HaNAC76*, in salt and drought tolerance. In our previous work, we have demonstrated *HaNAC76* responded to various abiotic stresses. Subsequently, *HaNAC76*-overexpressed and *HaNAC76-RNAi* vectors were constructed for gene transformation. After 72 h of co-cultivation following transient transformation, the inoculated seedlings showed elevated *HaNAC76* gene expression in overexpressing plants (OE) but reduced expression in interference plants (IE) (Figure 5a). Under normal culture conditions after *Agrobacterium* infection, there were no significant differences in the phenotypes of the *HaNAC76*-OE, the empty vector transformed plants (EV), and the *HaNAC76*-IE (Figure 5b). After 350 mM NaCl or 400 mM mannitol stress treatment for 3 d, respectively, the seedlings of the *HaNAC76*-OE remained healthy, however, the cotyledons of *HaNAC76*-IE exhibited more browning and necrosis (Figure 5b). Histochemical staining with DAB, NBT and Evans Blue, and physiological parameter measurements also showed *HaNAC76*-OE had significantly higher antioxidant enzyme activities and proline content coupled with lower MDA levels and reduced cell membrane damage. In contrast, *HaNAC76*-IE displayed opposite physiological responses (Figure 5c–h).

**Figure 6:**
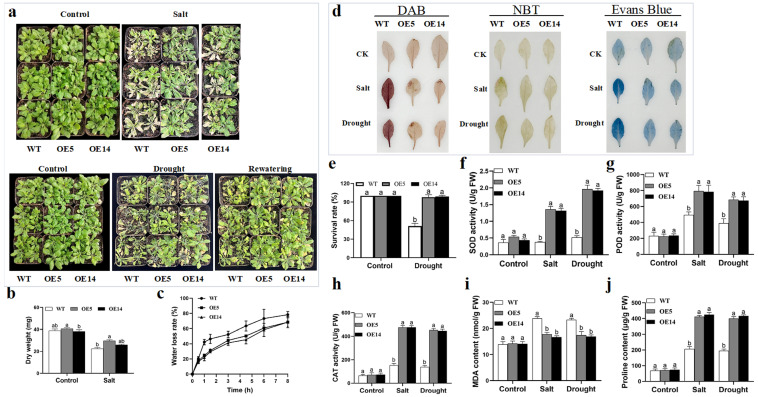
Identification of resistance in *HaNAC76* transgenic *Arabidopsis* under salt and drought stress. (**a**) Phenotypes of the WT and transgenic *Arabidopsis* plants exposed to 300 mM NaCl for 7 d and subjected to 10 d of drought, followed by 5 d of rehydration. (**b**) Dry weight measurements of the WT and transgenic *Arabidopsis* plants under stress conditions. (**c**) Rate of water loss in detached leaves of the WT and transgenic *Arabidopsis* plants. (**d**) DAB, NBT, and Evans Blue staining of the WT and transgenic *Arabidopsis* plants under stress conditions. (**e**) Survival rates of the WT and transgenic *Arabidopsis* during rehydration after drought treatments. (**f**–**j**) Physiological indices examined in the WT and *HaNAC76* transgenic *Arabidopsis* under salt and drought stress, including SOD activity (**f**), POD activity (**g**), CAT activity (**h**), MDA (**i**), and proline content (**j**). The Tukey’s comparison test was employed to analyze intergroup differences in physiological indices, with different lowercase letters indicating significant differences at the *p* < 0.05 level.

*HaNAC76* was heterologously expressed in *Arabidopsis thaliana*, generating homozygous single copy lines OE5 and OE14 with high relative expression. For stress assays, four-week-old plants were treated with either 300 mM NaCl or exposed to 10 days of natural drought. *HaNAC76*-OE displayed markedly enhanced growth vigor, and the aboveground dry weight was significantly higher compared to the WT (Figure 6a,b). After 5 d of rehydration, *HaNAC76* OE5 and OE14 showed complete revitalization, whereas the survival rate of the WT was only 50% (Figure 6a,e). Additionally, the water loss rate in detached leaves of *HaNAC76*-OE was lower than that of the WT (Figure 6c). Besides, the trends observed in histochemical staining and physiology indices in the transformation system of *Arabidopsis thaliana* were consistent with the results from the sunflower transient platform assay (Figure 6d,f–j). These indicates that *HaNAC76* could enhance salt and drought tolerance in both sunflower and *Arabidopsis* by improving the plants’ antioxidant and osmoregulatory capacities. Collectively, these experimental data further validate the reliability of the sunflower transient transformation platform for rapid gene functional analysis.

## 3. Discussion

*Agrobacterium* is widely utilized for transient expression in plants; however, several factors can influence transformation efficiency, including the strains, treatment methods, selection of explants, *Agrobacterium* concentration, infection time [35,36,37], and so on. The *Agrobacterium* strain GV3101 has demonstrated high infectivity in various plants, including apple (*Malus pumila*), poplar (*Populus*), tomato (*Solanum lycopersicum*), and safflower (*Carthamus tinctorius*) [29,38,39,40]. In this study, transformation conditions were optimized using three methods—infiltration, injection, and ultrasonic-vacuum—to establish a transient transformation system for sunflower with a high transformation rate using *Agrobacterium* strain GV3101. Furthermore, the selection of explants is critical to enhancing the efficiency of *Agrobacterium*-mediated transformation [41,42]. Three-day-old hydroponically cultivated seedlings with transient transformation by infiltration had normal growth, and the transformation efficiency of the GUS assay was up to 90%, which could be used for the subsequent identification of the gene function (Figure 1). For the injection method, sunflower seedlings grown for 7 to 9 days are optimal for injection (Figure 2d). Exogenous gene expression efficiency is significantly reduced in both newly expanded and senescing cotyledons. *Agrobacterium* density and infection time also affect the transient transformation efficiency in plants [34]. A high concentration of *Agrobacterium* can cause serious damage to the plant, while an insufficient concentration does not ensure enough *Agrobacterium* infection to the plant. Meanwhile, it takes time for exogenous genes to enter plant cells and be expressed. For optimal transient transformation, *Agrobacterium tumefaciens* strains EHA105 or GV3101 should be cultured to an OD_600_ of 0.8–1.2, followed by 3–5 days of co-cultivation [43,44,45]. Our experimental data specifically determined that an *Agrobacterium* concentration with an OD_600_ of 0.8 (Figure 1c, Figure 2d and Figure 3a) with three-day co-cultivation (Figure 2c) achieved maximal transformation efficiency in sunflower.

Surfactants are compounds that significantly reduce the surface tension at various gas−liquid interfaces, promoting *Agrobacterium* invasion and increasing plant cell permeability. Surfactants are commonly used to establish plant transient transformation systems including Silwet L-77, Tween-20, and Triton X-100. A study reported that the use of 0.04% Silwet L-77 improved the transient transformation of camphor tree (*Cinnamomum camphora*) [21]. In comparisons of cotton (*Gossypium hirsutum*) transient transformation using Tween-20 and Silwet L-77, Silwet L-77 significantly induced *GUS* gene expression [44]. Sunflower infiltration required the addition of 0.02% Silwet L-77 to the *Agrobacterium* suspension to improve transformation efficiency (Figure 1a and Figure 2a). Additionally, experimental results also suggest that Triton X-100 or Tween-20 may be more suitable for certain plant species, such as sweet wormwood (*Artemisia annua*) [46] and Chinese peony [44]. Therefore, screening for appropriate surfactants is essential.

Ultrasonic and vacuum infiltration can significantly increase the transient transformation efficiency in plants [47,48]. Ultrasonic can create a slight wound on the seedling surface, while vacuum filtration generates negative pressure to drive *Agrobacterium* into the plant, resulting in a significant increase in transient expression rate. Our results showed that with an ultrasonic duration at 40 kHz for 1 min and vacuum infiltration with 0.05 kPa for 5 to 10 min, sunflower seedlings achieved 100% transformation efficiency (Figure 3b,c).

Plant transient transformation generally has the highest level of gene expression at 2–4 d post-infection, after which there is a decline in both the number of expressing cells and the expression level per transformed cell. This kinetic trend is ultimately influenced by the host plant species [49]. We observed that the transcriptional levels of genes expressed in sunflower peaked at 2–5 d post-infection (Figure 4). This period can be utilized for subsequent studies such as investigating gene function and molecular interactions.

Transient transformation systems are valuable tools for analyzing gene function, molecular interactions, and subcellular localization. Tobacco remains the most widely employed species for this technology, routinely serving as a model for subcellular localization studies and molecular interaction assays [11]. Due to the limitations associated with heterologous gene expression, researchers employed a transient transformation system to identify the promoter activities of homologous genes *GhGPX1* and *GhGPX8*, as well as the subcellular localization of their corresponding proteins in cotton [43]. In addition, the *Agrobacterium*-mediated transient transformation method successfully detected the efficiency of sgRNA in the CRISPR systems of sorghum and birch [50,51]. The role of components of the ABA signaling pathway in response to drought was elucidated using a transient transformation system in mulberry seedlings [52]. ChIP-seq of AaHY5—a positive regulator of artemisinin biosynthesis—performed in a transient expression system uncovers the role of *AaWRKY14* in artemisinin biosyntheticregulation [53]. SCARECROW-like 32 (*ThSCL32*) gene transient overexpression conferred salt tolerance in *Tamarix hispida* and ThSCL32 can bind a conserved SBS motif within a transposon-derived *ThPHD3* promoter, establishing a direct regulatory module for salt adaptation of *Tamarix hispida* by ChIP-PCR with this plants that underwent transient gene transformation [54]. We also utilized *Agrobacterium*-mediated transient transformation in sunflower and stable transformation in *Arabidopsis* to confirm the function of the candidate gene *HaNAC76* in salt and drought tolerance (Figure 5 and Figure 6). In conclusion, the transient transformation system for sunflower is currently efficient and applicable for gene function validation and the investigation of molecular interactions.

## 4. Materials and Methods

### 4.1. Preparation of Experimental Materials and Treatment of Salt and Drought Stresses

Seeds of the oil sunflower backbone parent “Zaoaidatou” (ZADT) were provided by the Crop Research Institute, Xinjiang Uygur Autonomous Region Academy of Agricultural Sciences. The sunflower seedlings were cultivated in three distinct environments. For the infiltration method: the shelled seeds were sown on filter paper for germination and transferred to hydroponic containers with 1/2-strength Hoagland’s solution and germinated seedlings grew under low-light conditions (light intensity of 1000 lx, 25 °C/16 h light, and 23 °C/8 h dark) for 3 days. For the injection method: sunflower seeds were sown in nutrient soil and cultured in a greenhouse for 7 d (light intensity of 2000 lx, 28 °C/16 h light, and 26 °C/8 h dark). For the ultrasonic-vacuum method: the shelled seeds were sown in Petri dishes soaked with 1/2 MS and grown at 28 °C for 3 d in the dark.

Three-day-old hydroponically grown seedlings were transiently transformed and then maintained in culture for 3 d under normal growth conditions. Six-day-old hydroponic sunflower seedlings were incubated in a 1/2 Hoagland nutrient solution containing 350 mM NaCl or 400 mM mannitol (simulated drought stress) for 3 days. Stress phenotypes were observed, and the seedlings were collected, immediately frozen in liquid nitrogen and stored in a refrigerator at −80 °C for subsequent physiological analysis.

The *HaNAC76* gene was stably transformed into *Arabidopsis thaliana* Columbia-0 (WT) using the flower dip method. T_2_-generation *Arabidopsis thaliana* seeds with single-copy insertions were selected through kanamycin resistance screening. The growth conditions were set to a 16 h/8 h light/dark photoperiod at 22 °C in a growth room. Integration and expression analysis of the *HaNAC76* gene was performed using genomic PCR and qRT-PCR, and *Arabidopsis* lines with high expression were selected for 300 mM NaCl or exposed to 10 days of natural drought.

### 4.2. Agrobacterium-Mediated Transient Transformation in Sunflower

**Figure 7:**
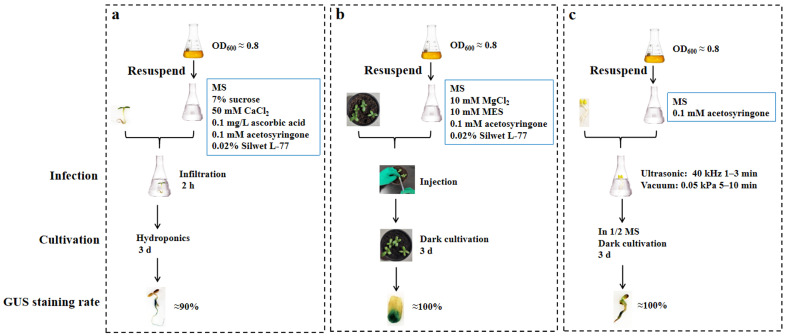
Flow chart of *Agrobacterium*-mediated transient transformation in sunflower by the three methods of Infiltration (**a**), Injection (**b**), Ultrasonic-vacuum (**c**).

Three methods of infiltration, injection, and ultrasonic-vacuum were employed for *Agrobacterium*-mediated transient transformation in sunflower. The detailed workflow was illustrated in Figure 7.

#### 4.2.1. Infiltration

The infiltration method was evaluated based on three factors: surfactant types (0.02% Silwet L-77 and 0.02% Triton X-100), *Agrobacterium* concentration (OD_600_: 0.4, 0.8, and 1.2), and infiltration time (1, 2, 4, 6, and 8 h).

#### 4.2.2. Cotyledonary Injection

The method of injecting cotyledons was optimized by four factors: surfactant types (0.02% Silwet L-77 and 0.02% Triton X-100), *Agrobacterium* concentration (OD_600_: 0.4, 0.8, and 1.2), seedling growth duration (5, 7, and 9 days), and dark cultivation time (1, 3, and 5 days).

#### 4.2.3. Ultrasonic-Vacuum Method

The ultrasonic-vacuum method was evaluated based on three factors: *Agrobacterium* concentration (OD_600_: 0.4, 0.8, and 1.2), ultrasonic time (1, 3, and 5 min), and vacuum time (1, 5, and 10 min).

To evaluate the impact of various factors on the transient transformation efficiency, a univariate experimental design was employed. Each experiment was repeated at least three times, with 10 seedlings per replicate. Transformation efficiency was calculated as the number of GUS-stained seedlings divided by the total number of seedlings. The data results are counted in Supplementary Table S1.

### 4.3. Detection of Transient Transformation Efficiency and Gene Expression Duration

After transformation using the three methods, the sunflower seedlings were transferred to their designated environmental conditions for cultivation. For the infiltration method, seedlings were subjected to GUS staining at 2, 4, and 6 days post-infiltration, while seedlings treated with the injection method and the ultrasonic-vacuum method were stained at 3, 6, and 9 days.

### 4.4. β-Glucuronidase (GUS) Staining

Seedlings in GUS staining solution were stained at 100 rpm in a shaker at 37 °C for 16 h in darkness. The staining solution was then washed away with water. The samples were rinsed with 50%, 70%, and 100% ethanol for 5 min each, and the excess liquid was discarded. The samples were subsequently soaked in 70% ethanol at 75 °C until all chlorophyll was removed. Sites stained blue indicated *GUS* expression.

### 4.5. Construction of Recombinant Plasmids

The vector pBI121-*GUS* was transformed into *Agrobacterium tumefaciens* strain GV3101. The open reading frame regions of *HaNAC76* containing 1650 bp removing stop code, was inserted downstream of the CaMV35S promoter in the binary expression vector pCAMBIA2300-*GFP*. Recombinant plasmids *HaNAC76*::*GFP* was constructed through homologous recombination. The plant interference expression vector used in this study was pCAMBIA2300-35S-Xi, purchased from Bio-Transduction Lab Co. Ltd. This vector contained an intron located between the 35S promoter and the NOS terminator. Through homologous recombination, the positive and negative strands of the specific interfering fragment of the *HaNAC76* gene were inserted at both ends of the intron in pCAMBIA2300-35S-Xi, resulting in the construction of the recombinant plasmid for the interference vector. The recombinant vector was transformed into *Agrobacterium tumefaciens* GV3101. The primers used for vector construction were listed in Supplementary Table S2.

### 4.6. qRT-PCR Experiment

Total RNA was extracted from 100 mg of the seedlings using the Plant RNA Kit (Omega BioTek, Norcross, GA, USA) according to the manufacturer’s instructions. Following DNaseI treatment, RNA was reverse-transcribed into cDNA using SuperScript™ II Reverse Transcriptase (Takara, Kusatsu, Japan) and a polyzai(dT)_18_ primer. qRT-PCR was performed on a CFX96 Touch Real-Time PCR System (Bio-Rad, Hercules, CA, USA), with three biological replicates for each sample. Relative gene expression was calculated using the 2^−ΔΔCT^ method [55]. *HaEF-1* and *HaActin* were used as internal reference genes, and the average expression of these two genes was calculated for analysis. Primer information for target genes (*GUS* and *HaNAC76*) used in the qRT-PCR experiments was provided in Supplementary Table S2.

### 4.7. Analysis of Physiological Indicators

*Arabidopsis* leaves (OE and WT) and cotyledons from transiently transgenic sunflower seedlings about the overexpressing plants (OE), the interference plants (IE), and the empty vector transformed plants (EV) were stained with 1 mg/mL Evans Blue, DAB, and NBT solution, followed by 2 h of light protection at 37 °C and boiled in anhydrous ethanol for 30 min to remove chlorophyll. SOD, POD, CAT, MDA, and Proline were measured using detection assay kits from the Beijing solarbio science technology Co., Ltd. (Beijing, China) based on the manufacturer’s instructions. Survival rate refers to the percentage of live plants in the total number.

The calculation method for the water loss rate of detached leaves was as follows. The detached leaves were placed on a tabletop. The initial fresh weight was recorded as W_o_, and the weight of the leaves was measured every 30 min as W_t_. The water loss rate was calculated using the formula: Water loss rate = 100% − (W_t_/W_o_).

### 4.8. Data Analysis and Graph Construction

Data analysis was performed using GraphPad Prism 8.0.2 and SPSS Statistics 20 (IBM, Armonk, NY, USA). Experimental results were assessed through ANOVA. Treatment differences were presented as means ± SD and were compared at a significance level of *p* < 0.05 using Tukey’s LSD test. Image processing was carried out using Adobe Photoshop software.

## 5. Conclusions

In this study, we successfully established an *Agrobacterium*-mediated transient transformation system in sunflower using three methods: infiltration, injection, and ultrasonic-vacuum. This system can be applied for gene function identification and mechanism elucidation. We comprehensively evaluated the effects of various factors on transformation efficiency, including surfactant types, *Agrobacterium* concentration, seedling age, infiltration time, co-culture time, ultrasonic time, and vacuum time. Our results indicated that the highest transformation rate of 90% in sunflower was achieved using the infiltration method. Moreover, transformation rates of 100% were obtained using the injection and ultrasonic-vacuum methods. Gene expression in the transiently transformed sunflower was sustained for at least 6 days. Furthermore, we successfully validated the salt and drought tolerance of the candidate gene *HaNAC76* in situ using the sunflower transient transformation system. The establishment of this system facilitates efficient research in the molecular field of sunflower.

## Data Availability

All data generated or analyzed during this study are included in this published article and its Supplementary Information Files.

## Supplementary Materials

The following supporting information can be downloaded at: https://www.mdpi.com/article/10.3390/plants14152412/s1, Table S1: The number of positive seedlings transformed transiently using different methods; Table S2: Summary of primer sequences.
