# Supplementary material for: Establishment of Agrobacterium-Mediated Transient Transformation System in Sunflower
Source: Plants (Basel). 2025 Aug 4;14(15):2412. doi: 10.3390/plants14152412 (PMC12349338; doi:10.3390/plants14152412)
Supplement: Supplementary file 1 [file plants-14-02412-s001.zip › plants-3761661 Supplementary Table S1.pdf]

Supplementary Table S1 The number of positive seedlings transformed transiently using different methods

Method	Condition	Gradients	Total number	Number of positive seedlings
Infiltration	surfactant type	Silwet L-77	10, 10, 10	9, 9, 9
		Triton X-100	10, 10, 10	7, 8, 7
	infiltration time (h)	1	10, 10, 10	7, 8, 8
		2	10, 10, 10	9, 9, 9
		4	10, 10, 10	8, 9, 7
	Agrobacterium concentration (OD_{600})	0.4	10, 10, 10	6, 5, 7
		0.8	10, 10, 10	9, 9, 9
		1.2	10, 10, 10	8, 9, 9
Injection	surfactant type	Silwet L-77	10, 10, 10	10, 10, 10
		Triton X-100	10, 10, 10	9, 10, 10
	Agrobacterium concentration (OD_{600})	0.4	10, 10, 10	10, 10, 10
		0.8	10, 10, 10	10, 10, 10
		1.2	10, 10, 10	10, 10, 10
	dark cultivation time (d)	1	10, 10, 10	10, 10, 9
		3	10, 10, 10	10, 10, 10
		5	10, 10, 10	9, 10, 9
	seedling growth stage (d)	5	10, 10, 10	10, 10, 10
		7	10, 10, 10	10, 10, 10
		9	10, 10, 10	10, 10, 10
Ultrasonic-vacuum	*Agrobacterium* concentration (OD_{600})	0.4	10, 10, 10	10, 10, 10
		0.8	10, 10, 10	10, 10, 10
		1.2	10, 10, 10	10, 10, 10
	Ultrasonication （40 kHz, min）	1	10, 10, 10	10, 10, 10
		3	10, 10, 10	9, 9, 9
		5	10, 10, 10	8, 9, 9
	Vacuum infiltration (0.05 kPa, min)	1	10, 10, 10	9, 9, 9
		5	10, 10, 10	10, 10, 10
		10	10, 10, 10	10, 10, 10
