# Supplementary material for: Establishment of Agrobacterium-Mediated Transient Transformation System in Sunflower
Source: Plants (Basel). 2025 Aug 4;14(15):2412. doi: 10.3390/plants14152412 (PMC12349338; doi:10.3390/plants14152412)
Supplement: Supplementary file 1 [file plants-14-02412-s001.zip › plants-3761661 Supplementary Table S2.pdf]

Supplementary Table S2 Summary of primer sequences

Primer name	Primer sequence (5'-3')	Aim of amplifying genes with primers
qRT-*HaActin*-F	CCCATCCATTGTCCACCGAA	qRT-PCR of *HaActin*
qRT-*HaActin*-R	CCGCATACCGATGCATGAAC	
qRT-*HaEF-1*-F	AGCCTCTTCGTCTCCCACTTC	qRT-PCR of *HaEF-1*
qRT-*HaEF-1*-R	ACCATACCGGGCTTGATCAC	
qRT-*GUS*-F	AGACTGTAACCACGCGTCTG	qRT-PCR of *GUS*
qRT-*GUS*-R	TGTCTGGCTTTTGGCTGTGA	
HaNAC76-F	GGACGAGCTCGGTACCCGGGATGGAAGTCGTGCCAGTAAA	The pCAMBIA2300-35S-GFP-*HaNAC76* vector was constructed
HaNAC76-R	CTCACCATGTCGACTCTAGAGTCATCTCTCACTACAAAAC	
HaNAC76-RNAi-S-F	TGGAGAGAACACGGGGGACGGATCCCCACCCGTACATTTAGATTCG	The pCAMBIA2300-35Si-X-*HaNAC76*-s vector was constructed
HaNAC76-RNAi-S-R	CTTAATTACCCTCTACTAGTCGACCCGTCTGATTCGCCACTATAATC	
HaNAC76-RNAi-A-F	GTAATCAATTGTTAGGATTTCTAGACCGTCTGATTCGCCACTATAATC	The pCAMBIA2300-35Si-X-*HaNAC76*-a vector was constructed
HaNAC76-RNAi-A-R	GAAATTCGAGCTCAGATCTGGTACCCCACCCGTACATTTAGATTCG	

Note: The Tm values for each primers were: 57°C for *HaActin*; 58°C for *HaEF-1*; 58°C for *GUS*; 60°C for *HaNAC76*.
